# The transferability and validity of a population-level simulation model for the economic evaluation of interventions in diabetes: the MICADO model

**DOI:** 10.1007/s00592-022-01891-2

**Published:** 2022-04-21

**Authors:** Sajad Emamipour, Eva Pagano, Daniela Di Cuonzo, Stefan R. A. Konings, Amber A. van der Heijden, Petra Elders, Joline W. J. Beulens, Jose Leal, Talitha L. Feenstra

**Affiliations:** 1 Department of Clinical Pharmacy and Pharmacology, University Medical Center Groningen, University of Groningen, Groningen, The Netherlands; 2 Unit of Clinical Epidemiology, “Città della Salute e della Scienza” Hospital and CPO Piemonte, Turin, Italy; 3 Department of Psychiatry, Interdisciplinary Center Psychopathology and Emotion Regulation (ICPE), University Medical Center Groningen, University of Groningen, Groningen, The Netherlands; 4 Department of General Practice, Amsterdam UMC, Location VUMC, Amsterdam Public Health Institute, Amsterdam, The Netherlands; 5 Department of Epidemiology and Data Science, Amsterdam UMC, Location VUMC, Amsterdam Public Health Institute, Amsterdam, The Netherlands; 6 Nuffield Department of Population Health, Health Economics Research Centre, University of Oxford, Oxford, UK; 7 Faculty of Science and Engineering, Groningen Research Institute of Pharmacy, University of Groningen, Groningen, The Netherlands; 8 National Institute for Public Health and the Environment (RIVM), Bilthoven, The Netherlands

**Keywords:** Transferability, Diabetes complications, Cardiovascular disease, Health economics

## Abstract

**Aims:**

Valid health economic models are essential to inform the adoption and reimbursement of therapies for diabetes mellitus. Often existing health economic models are applied in other countries and settings than those where they were developed. This practice requires assessing the transferability of a model developed from one setting to another. We evaluate the transferability of the MICADO model, developed for the Dutch 2007 setting, in two different settings using a range of adjustment steps. MICADO predicts micro- and macrovascular events at the population level.

**Methods:**

MICADO simulation results were compared to observed events in an Italian 2000–2015 cohort (Casale Monferrato Survey [CMS]) and in a Dutch 2008–2019 (Hoorn Diabetes Care Center [DCS]) cohort after adjusting the demographic characteristics. Additional adjustments were performed to: (1) risk factors prevalence at baseline, (2) prevalence of complications, and (3) all-cause mortality risks by age and sex. Model validity was assessed by mean average percentage error (MAPE) of cumulative incidences over 10 years of follow-up, where lower values mean better accuracy.

**Results:**

For mortality, MAPE was lower for CMS compared to DCS (0.38 vs. 0.70 following demographic adjustment) and adjustment step 3 improved it to 0.20 in CMS, whereas step 2 showed best results in DCS (0.65). MAPE for heart failure and stroke in DCS were 0.11 and 0.22, respectively, while for CMS was 0.42 and 0.41.

**Conclusions:**

The transferability of the MICADO model varied by event and per cohort. Additional adjustments improved prediction of events for MICADO. To ensure a valid model in a new setting it is imperative to assess the impact of adjustments in terms of model accuracy, even when this involves the same country, but a new time period.

**Supplementary Information:**

The online version contains supplementary material available at 10.1007/s00592-022-01891-2.

## Introduction

The international diabetes federation (IDF) estimated that the number of adults (aged 20–79 years) with diabetes will increase from 463 million in 2019 to 700 million in 2045 [1]. Preventing diabetes complications through effective and cost-effective care is therefore an important task of health policy makers [2]. Health economic decision models are used to inform health policy decision making concerning diabetes management. These models allow to simulate the natural history of disease and translate the impact of treatments from short-term outcomes as measured in experimental studies to decision-relevant outcomes such as (quality-adjusted) life expectancy [3]. Such models are usually initially developed for a specific jurisdiction, usually a country. Furthermore, the use of input data from a certain time period implies a certain base year. However, subsequent model applications may require transfer to other settings and time periods. Diabetes health economic decision models are mainly used to estimate the cost-effectiveness of treatments. Several health economic decision models of diabetes have been developed and validated in the past decades [2], including MICADO (Modeling Integrated Care for Diabetes based on Observational data), which was developed for the Netherlands.

The MICADO model projects the prevalence and effect of both micro- and macrovascular complications in populations with diabetes. This model was developed to reflect the diabetes population in the Netherlands in 2007, and has been validated both internally and externally [3]. This model participated in several Mount Hood Challenges for cross-validation [4]. The structure of MICADO was based on the Dutch Chronic Disease Model [5].

It is common practice to transfer health economic models to the setting of relevance to the decision-maker when evaluating new treatments in that setting. In such cases, decision-makers want to know if a model is valid for use in their own country and time period, and whether it needs to be adjusted to achieve this. Assessing and enhancing the transferability of health economic decision models is challenging [6, 7]. Health economic models comprise several correlated parameters and simulate a variety of outcomes, from event-specific rates to (quality-adjusted) life expectancy. Some deliberate examples of health economic models being transferred to a new setting have been published [8, 9], while many more exist, usually as part of health technology assessment dossiers. However, previous studies rarely assessed the validity of the transferred model predictions and often focused on producing cost-effectiveness results in the new setting, without paying much attention to revalidating the transferred model in its new setting. Most often, adjustments focus on replicating the demographic characteristics of the new setting, as well as using country-specific utilities and costs. This lack of explicit attention for re-validating a transferred model and assessing more fully its transferability may relate to the rather qualitative approach taken by most transferability tools.

In this paper, we evaluate the transferability of the diabetes simulation part of MICADO to (1) a more contemporary Dutch setting and (2) an Italian setting by explicit and elaborate validation against empirical data. We aim to assess its transferability in an objective way and infer guidance regarding the adjustment steps needed for any health economic decision model of diabetes to be transferred to a new setting.

## Methods

We assessed the transferability by re-validating the MICADO model using a Dutch [10] (Hoorn Diabetes Care System [DCS]) and an Italian [11, 12] (Casale Monferrato Survey [CMS]) diabetes cohort. Existing transferability checklists were scrutinized to guide our adjustment steps. The characteristics of each cohort at baseline are presented in Table 1. Details on data selection and imputation are presented in Supplementary Material (see sections “Data selection” and “Missing values”). Model results were compared to empirical observations to assess the validity of model transfer and the additional value of adjustment steps.

**Table 1:** Characteristics of two study cohorts, at baseline

Characteristic	CMS (Italian)	DCS (Dutch)
*N*	1931	5188
Male (%)	48.9	55.6
Mean (SD) age (years)	67.8 (10.3)	64.8 (11.1)
Mean (SD) diabetes duration (years)	10.9 (8.0)	6.8 (5.9)
Mean (SD) BMI (kg/m^2^)	28.5 (5.0)	30.3 (5.5)
Mean (SD) HbA1c (mmol/mol)	53 (19)	50 (11)
Mean (SD) HbA1c (%)	7.0 (1.7)	6.7 (1.0)
Current smoker (%)	14.9	18.6
Mean (SD) HDL (mmol/L)	1.4 (0.4)	1.2 (0.3)
Mean (SD) LDL (mmol/L)	3.3 (0.9)	2.6 (0.9)
Mean total cholesterol (SD), (mmol/L)	4.8 (1.0)	4.6 (1.3)
Mean (SD) systolic blood pressure (mmHg)	146.1 (16.4)	142.1 (20.1)
History of MI (%)	7.9	7.7
History of stroke (%)	6.7	6.0
History of CHF (%)	1.9	2.7

*CMS* Casale monferrato, *DCS* Hoorn diabetes care system, *BMI* body mass index, *HbA1c* glycated hemoglobin, *LDL* low-density lipoproteins, *HDL* high-density lipoproteins, *SD* standard deviation, *MI* myocardial infarction, *CHF* chronic heart failure

### The MICADO model

MICADO is a state-transition model with a cycle length of one year. The structure of the model is based on the multistate life table method [3, 13]. The transition rates hence depend on age category, sex, and category of HbA1c-level, as well as on further risk factors by categories (smoking status; Body Mass Index, BMI; systolic blood pressure, SBP; and total cholesterol). Risks of cardiovascular complications in diabetes were modeled using age, sex, and risk factor, that also vary depending on pre-existing events, which were derived from a literature review. The detailed structure of the model has been published previously [14, 15]. Based on the risk factors and pre-existing events, the model simulates at the population level the incidence and prevalence of microvascular (diabetic foot, nephropathy and retinopathy) and macrovascular (myocardial infarction [MI], chronic heart failure [CHF] and stroke) diabetes-related complications, and provides estimates for all-cause mortality (by age and sex), complication-related mortality, costs of complications, and QALYs. In the current study, we focused on macrovascular events and all-cause mortality. We report more details on MICADO inputs in Supplementary Materials (see section “Input data into MICADO”).

### Selection of transferability items

A previous review identified seven unique checklists, flowcharts, criteria and tools to assess the geographic transferability of health technology assessments [16]. We reviewed the seven transferability checklists and tools for items referring specifically to the disease simulation part of decision models [16]. Common across the checklists, is the reliance on expert opinion to assess the transferability of a model and the need for any adjustments.

The transfer items identified in the checklists, concerning decision models, were as follows: age and sex, health status and severity of disease, life expectancy, complication rates, socio-economic and educational status. Based on these transfer items, MICADO met the criteria of being capable of transfer across different settings. Hence, to transfer MICADO, we adjusted the model for each of these items in discrete steps to the CMS and DCS settings (see Supplementary material, section “Assessing transferability of MICADO” for more detail). For comparison, our base case adjustment reflects a minimal adjustment, consisting of using the demographic characteristics of the new setting. We then evaluated the impact of each additional adjustment step (see Table 2) on the accuracy/validity of the model by comparing outcome predictions to actual observed events over time. The main reason for such order was that the complexity of model would increase by each step and needs more data for adjusting the model. The adjustments started with adjusting demography (base case) and ended with adjusting mortality rate based on age and sex (adjustment step 3).

**Table 2:** Model adjustment steps

Adjustment step	Adjustment
Base case	Adjusted age and sex of the modeled population (demography)
1	Adjusted demography and distribution of risk factors at baseline (BMI, smoking, TC, SBP and HbA1c)
2	Adjusted demography, risk factors and prevalence of pre-existing events at baseline (MI, CHF and stroke)
3	Adjusted demography, risk factors, events at baseline, and the general population mortality rate by age and sex

*BMI* Body mass index, *TC* total cholesterol, *SBP* systolic blood pressure, *HbA1c* glycated hemoglobin, *MI* myocardial infarction, *CHF* chronic heart failure

### Outcomes to be validated

Our analysis focused on the incidence of macrovascular diabetes-related outcomes (MI, CHF and stroke) and all-cause mortality. The macrovascular outcomes were defined according to the International Classification Codes (ICD9 and ICD10) listed in Table S5. All-cause mortality was obtained from national death registries in each country.

### Model validity and sensitivity analyses

Model validity was performed by comparing MICADO predictions with the mean and 95% CI of the observed cumulative incidences in each cohort at 10 years of follow-up. MICADO was judged to be well-calibrated if the model simulated cumulative event rates were within the 95% CI of the observed cumulative incidence [17, 18]. We also calculated the mean absolute percentage error (MAPE), the mean absolute error (MAE) and the root-mean-squared error (RMSE) to get a quantitative estimate of the calibration and compare the fit of each adjustment step [19]. Setting a threshold for these statistics was not possible, because there is no a global agreement on which value should be considered as a good fit. Instead, we compared all statistics (MAPE, MAE and RSME) for every outcome following each adjustment step. The adjustment that produced the lowest values was judged to have the best fit for a given outcome. For more details see the sections “Model validity” and “Sensitivity analyses” in Supplementary Materials.

## Results

### Model outcomes per adjustment step and calibration in the large

**Fig. 1:**
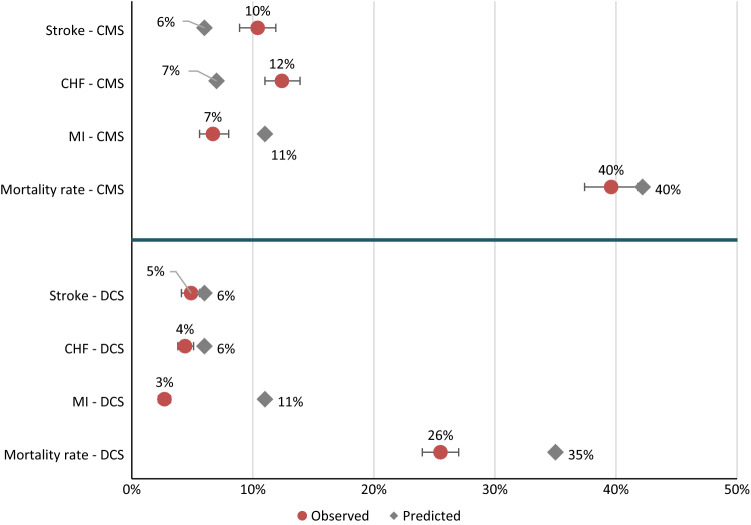
The observed versus predicted at year 10. CMS: Casale Monferrato Survey; DCS: Hoorn Diabetes Care System; MI: Myocardial infarction; CHF: Chronic heart failure

At 10 years of follow-up, the overall mortality was higher in the CMS cohort compared to DCS (40% vs. 26%) (Fig. 1). The three cardiovascular outcomes (MI, CHF and stroke) were also higher in the CMS cohort compared to DCS during the same period.

**Fig. 2:**
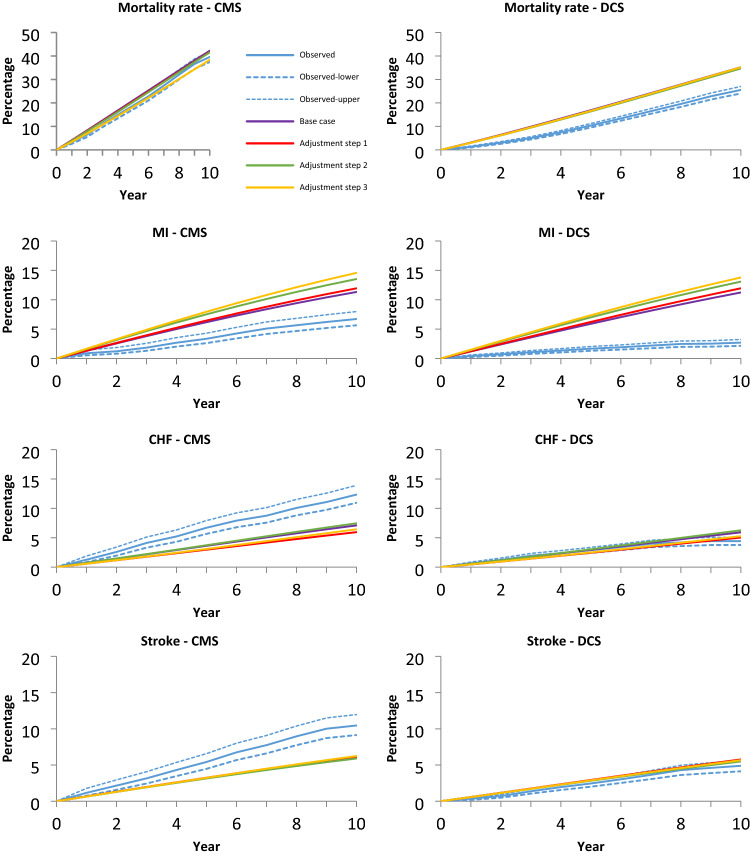
Observed events vs. the predicted events by model in different adjustment step. CMS: Casale Monferrato Survey; DCS: Hoorn Diabetes Care System; MI: Myocardial infarction; CHF: Chronic heart failure

Figure 2 shows the predicted and observed cumulative incidence of each outcome for each adjustment step over 10 years of follow-up in both cohorts, starting with base case adjustments and adding up to 3 adjustments to the target cohort. In all adjustment steps, for CMS, MICADO showed a good prediction for mortality, but underpredicted stroke and CHF while overpredicting MI. In contrast, for DCS, MICADO showed a good prediction for CHF and stroke but overpredicted mortality and MI.

MICADO overpredicted mortality in the DCS cohort and the impact of different adjustments was limited with model predictions outside the 95% CI. For CMS, further adjustments to the base case, resulted in a decrease in mortality rate (42 vs. 38% for the base case and step 3 adjustments), and all of predicted mortalities were in the 95% CI.

At 10 years, the incidence of MI was 7% (95%CI: 6–8%) and 3% (95%CI: 2–3%) in the CMS and DCS cohorts, respectively. However, MICADO predicted MI to be 11% in both cohorts. The predicted event rate of MI increased with more adjustments (14% to 15%) resulting in more over-prediction in both cohorts. MICADO underpredicted CHF and stroke in CMS, while in DCS the predicted values were close to observed for CHF and for stroke they were within the 95% CI (see Tables S7-S10).

The largest variation in MAPE between different adjustment steps was in MI for both cohorts (0.77–1.27 in CMS and 2.58–3.45 in DCS for the base case and step 3 adjustments, respectively). For the other outcomes for different adjustments, MAPE varied at most from 0.11 to 0.70.

### Impact of adjustments on model transferability

Table 3 lists which adjustment step resulted in the lowest values of MAPE (Table S11 shows for MAE and RMSE) by outcome and cohort over a 10 year time horizon compared to the base case. For CMS, adjustment step 3 improved MAPE for mortality by 48% (observed versus prediction: 40 vs. 38%). For DCS, the highest MAPE improvement was 8% for the mortality, following adjustment step 2 (observed versus prediction: 26 vs. 35%). For both cohorts, additional adjustments did not improve MAPE for MI compared to the base case adjustment. In CMS, adjustment step 2 resulted in the lowest MAPE for CHF (0.42), while adjustment step 1 worked best for stroke (0.41), improving model fit by 4% and 3%, respectively, compared to base case adjustment. In DCS, base case adjustment resulted in the lowest MAPE for CHF (0.11), while adjustment step 2 was best for stroke (MAPE: 0.22). For the results of subgroups and the sensitivity analyses please see Supplementary Materials sections “Results of subgroup analyses” and “Results of sensitivity analyses.”

**Table 3:** The adjustment steps with the lowest MAPE and the percentage of decrease in the MAPE relative to the base case adjustment and the observed versus predicted at year 10

Outcome	CMS						DCS					
	Observed (%)	95% CI (%)	Predicted (%)	AS	MAPE^*^, (%)	Change (%)	Observed (%)	95% CI (%)	Predicted (%)	AS	MAPE^*^ (%)	Change (%)
Overall mortality	40	37–42	38	3	20	48	26	24–27	35	2	65	8
MI	7	6–8	11	BC	77	–	3	2–3	11	BC	258	–
CHF	12	11–14	8	2	42	4	4	4–5	6	BC	11	–
Stroke	10	9–12	6	1	41	3	5	4–6	6	2	22	7

*CMS* Casale monferrato survey, *DCS* hoorn diabetes care system, *CI* confidence interval, *AS* adjustment step, *MAPE* mean absolute percentage error, *MI* myocardial infarction, *CHF* chronic heart failure, BC base case

*The lower values mean a better fit

## Discussion

It is important to assess the transferability of diabetes simulation models and validate transferred models before using them in different settings with confidence. After adjusting for demography, the MICADO model predicted mortality rates close to the observed values for the Italian cohort but it overestimated the rates of MI and underestimated the rates of stroke and CHF. In the Dutch cohort, the MICADO model overestimated the mortality and MI rates but showed a good fit for CHF and stroke. Additional adjustments improved the model fit, pointing at inadequacy of adjusting demographic characteristics only, without proper validation against empirical data from the new setting.

The MICADO model was developed based on Dutch data on mortality rates by age and sex, and the prevalence of events in 2007 [14]. While the Italian CMS cohort consisted of the individuals followed from 2000 to 2017, the Dutch DCS cohort had a follow-up from 2008 till 2019. In regression models, cross-validation is usually performed in regression analysis or prediction modeling studies to reduce overfitting. However, in the current study, we did not perform a regression analysis or fit a prediction model. Rather we applied an existing health economic simulation model (MICADO) to two independent cohorts. Since MICADO was not developed based on the two cohorts, overfitting is not relevant here. Rather, each adjustment step made while applying the model to the independent cohorts can be seen as external validation. The variation in the model accuracy concerning mortality across the cohorts might be explained by differences in the time periods covered by the model and cohorts. Moreover, the observed mortality rate of the Dutch diabetes cohort was almost 30% lower than the Italian cohort, suggesting that the Dutch cohort consisted of a better controlled diabetes population. In a previous study which compared the diabetes type 2 treatment across European countries, they reported that the guidelines for intensification of treatment (by adding a sulfonylurea) in Italy are less strict than the Netherlands [20]. Additionally, the diabetes care program in the DCS was structured and centralized with annual elaborate check-ups by a diabetes nurse [10].

Our findings show that model adjustments recommended in transferability checklists have limited impact in model predictions and the overall fit of MICADO across the two cohorts. For MI, more extensive adjustments even led to an increase in the MAPE. No single adjustment was clearly the best performing in both settings and across all outcomes. The most complex adjustments did not result necessarily in higher accuracy. However, the MICADO model met the checklist criteria for a transferable model and we were able to make several of the recommended adjustments to new settings. This highlights the potential limitations of basing the criteria in transferability checklists on expert opinion without formal assessments of model accuracy in the new setting. Validation against empirical data is an essential step in the transfer of a health economic diabetes model to a new setting. As pointed out in our study, validation and uncertainty were among the most important aspects of the ISPOR modeling good research practice [21]. To do so, we followed the common practice to measure the relative difference in the predicted against observed cumulative point estimates [18, 22].

Comparing the transferability of the MICADO model with previous transferability studies in type 2 diabetes modeling is challenging. We identified two studies, one assessing the transferability of the Building, Relating, Assessing, and Validating Outcomes (BRAVO) risk engine[23] (a US-based model) and another assessing the UKPDS Outcomes Model (UKPDS-OM) [24] (a UK-based model). The BRAVO model showed significant improvement of prediction accuracy for different global settings (the United States, Europe, Latin America, Africa, Asia) after re-calibration of the hazard ratios of MI, stroke, CHF, angina, revascularization and all-cause mortality [25]. The UKPDS-OM was transferred to an Australian population by re-estimating the model equations for predicting mortality of diabetes-related complications using a large local diabetes dataset. Similar to BRAVO, they reported a significant improvement on model fit following re-calibration [26]. Similar to our findings, these studies concluded that adjusting the model for the cohort-specific characteristics further improved the prediction accuracy and the validation of outcomes is required.

The DCS and CMS cohorts were used previously to assess the validity of version 2 of the UKPDS-OM [27]. The approach used in that work, coincided with our adjustment step 2 (i.e., adjusting for demography, risk factors and pre-existing events). However, no adjustment of mortality was performed, i.e., our step 3. This was due to the complexity of the UKPDS-OM, which uses 15 different connected prediction functions to estimate outcomes, including all-cause mortality [28]. Although MICADO and UKPD-OM have a different structure and were built using different input data, the performance of these two models on the Italian CMS and the Dutch DCS cohorts were quite similar. Both models predicted a mortality rate in CMS close to observed values but for DCS, they overestimated mortality. MI was overestimated by both models in these two cohorts. Both models predicted CHF and stroke in DCS close to observed values but overestimated them for CMS. This provides further support to the period effects as a partial explanation for poor performance of both models regarding MI and mortality. Also, the difference between population characteristics in Italy and the Netherlands might be another explanation (such as higher mortality rate in the Italian cohort).

For single outcome/equation prediction models, re-calibration of intercepts and the coefficients are routinely performed after transfer to a new setting [29]. However, re-calibration of decision models is more complex as their baseline risk of events is not informed by a single parameter, like the intercept or baseline hazard in a single risk prediction model. Many current applications only partially adjust population characteristics and seldom validate the decision model in the new setting. Re-calibration is a complex task requiring adjusting parameters across several interconnected equations and checking their joint impact on model predictions. Hence, we did not perform re-calibration, i.e., adjustment/re-estimation of risk equations, but rather adapted MICADO by changing the characteristics of the simulated population to match those of the validation cohort as well as updating general mortality rates to those more suitable for each validation setting. However, the adjustment of individual risk equations may be justifiable when using diabetes models to simulate the risks of MI, CHF and stroke in contemporary cohorts similar to the CMS or DCS.

Our work is not without limitations. We did not adjust relative risks of diabetic complications and risk factors in our adjustment steps. For MICADO, these model parameters were derived from reviews of international literature [30]. Furthermore, we did not adjust for the incidence and case fatality risk of complications, and the transitions of risk factors. The reasons for not doing so were absence of data in the CMS cohort. However, our sensitivity analyses showed that different transition rates had little impact on the model predictions. Socio-economic status was another parameter recommended for adjustment in transferability checklists. However, the MICADO model only allowed for adjustment for patient’s age, sex, risk factor levels and pre-existing events. Finally, the MICADO model is a cohort level state-transition model while many diabetes simulation models consist of patient-level state transition models, or discrete event simulation models [19, 24, 31, 32]. Our findings and adjustment steps may be more relevant to other cohort models [33–39], than to patient level models.

Strengths of this study were the use of two relatively large cohorts from different settings allowing evaluating transferability of a Dutch model to a setting outside the Netherlands, covering the same period as the original model, and a more contemporary Dutch setting. Another strength is that the adjustments needed to transfer the model to the new settings, were systematically performed in a stepwise way, assessing performance of the model for each adjustment step.

In conclusion, the MICADO model showed good transferability for mortality in the Italian CMS cohort, and for CHF and stroke in the Dutch DCS cohort. We showed that additional adjustments, especially regarding the baseline distribution of risk factors in the population, improved the prediction accuracy of mortality, MI and stroke for MICADO. However, our findings suggest that the most complex adjustment steps did not always result in the most accurate model. Therefore, simply performing the model adjustments as suggested in transferability checklists do not necessarily translate into a valid model in a new setting. This highlights the need for model validation using observed data rather than relying solely on expert opinion to assess its transferability. After this has been established, additional adjustments on other model elements needed for economic evaluations such as costs and utilities can be performed.

## Supplementary Information

Below is the link to the electronic supplementary material.

Supplementary file1 (DOCX 235 KB)

## Data Availability

Restrictions apply to the availability of data generated or analyzed during this study to preserve patient confidentiality or because they were used under license. The corresponding author will on request detail the restrictions and any conditions under which access to some data may be provided.
