# Supplementary material for: The transferability and validity of a population-level simulation model for the economic evaluation of interventions in diabetes: the MICADO model
Source: Acta Diabetol. 2022 Apr 21;59(7):949–57. doi: 10.1007/s00592-022-01891-2 (PMC9156453; doi:10.1007/s00592-022-01891-2)
Supplement: Supplementary file 1 — Supplementary file1 (DOCX 235 KB) [file 592_2022_1891_MOESM1_ESM.docx]

**Supplementary materials**

*Data selection*

Casale Monferrato Survey (CMS)

In 1988, a survey was started in the region of Casale Monferrato, northwest Italy, to assess the prevalence of known diabetes in individuals [1]. In 2000, a new survey was performed which included the alive members of the original survey who were still living in Casale Monferrato (N=860) in addition to all new diagnoses of type 2 diabetes (N=2389) identified through diabetes clinics and from administrative data records [2].

For the current study, a cohort of 1931 patients (out of 3249 patients) was used that had complete data on a core set of risk factors as well as on outcomes. Figure S1 shows the flowchart of selected individuals. History of MI and history of stroke were partially missing. Vital status was last updated at the end of 2017. Data on risk factor values, including BMI, HbA1c and blood pressure were measured at baseline (2000). For the entire period of 2000-2017, outcomes during follow-up were classified according to ICD-9-CM codes

Hoorn Diabetes Care System (DCS) cohort

The DCS cohort in the Netherlands is a dynamic primary care registry of individuals diagnosed with type 2 diabetes [3]. From the start of the study in 1998, people with type 2 diabetes treated by primary care physicians in a sub-region of the Netherlands (West-Friesland) could enter the study and participate in centralized diabetes care. Since 2008, a random subsample was invited for the DCS biobank (N= 5946) [4]. Participants in this biobank subsample were asked for consent to link with medical records, to enable validation of self-reported information on events with hospital records. Causes of death were ascertained from national death records and last updated at the end of 2019. All outcomes were recorded using ICD-9 definitions and carefully aligned with the definitions of CMS and MICADO. For the current study, a sample of N=5188 patients was included, with complete data on core variables at baseline, and at least 5 years of follow-up data available, implying year of entry before 2013. The flowchart of selected individuals is shown in Figure S2. Variables include annual measurements of risk factor values, events and vital status.

*Missing values*

The missing values of risk factors were assumed to be missing at random (MAR) and imputed. The imputation method has been extensively explained in a previous publication [5]. In short, the percentage of individuals with missing values at baseline was 24% and 3.2% for CMS and DCS, respectively. Multiple imputation [5] was used to predict the missing values at patient level. The number of imputed datasets were 25 for both cohorts. For continuous variables the average and for binary variables the most frequent values were used for the final imputed values.


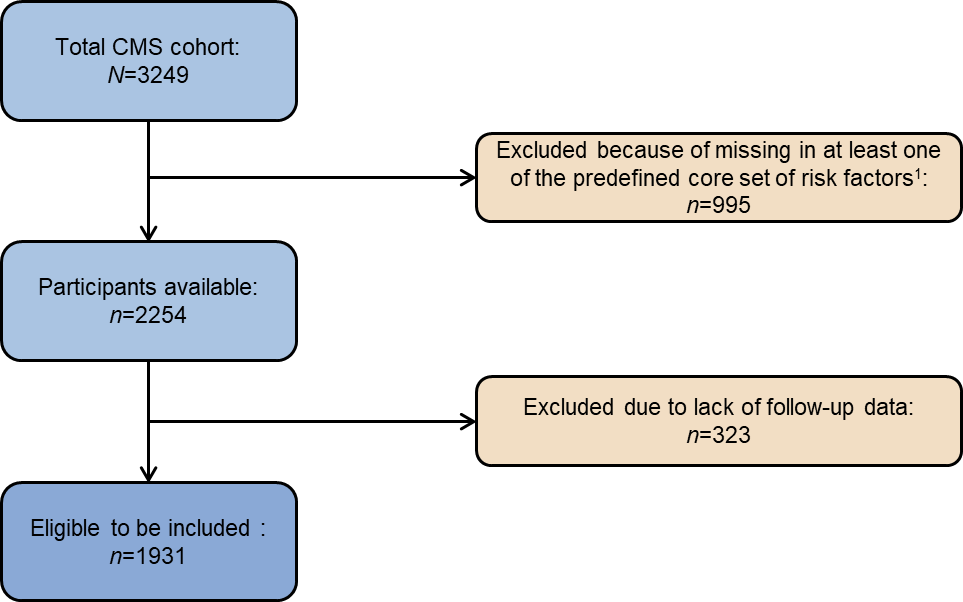


Figure S1: The flowchart of individual selection in Casale Monferrato Survey (CMS)

^1^Predefined core set of risk factors: sex; age; duration of diabetes (years); BMI; smoking status (current smoker or not); total, HDL and LDL cholesterol; systolic blood pressure; glycated haemoglobin (HbA1c); and estimated glomerular filtration rate (eGFR)


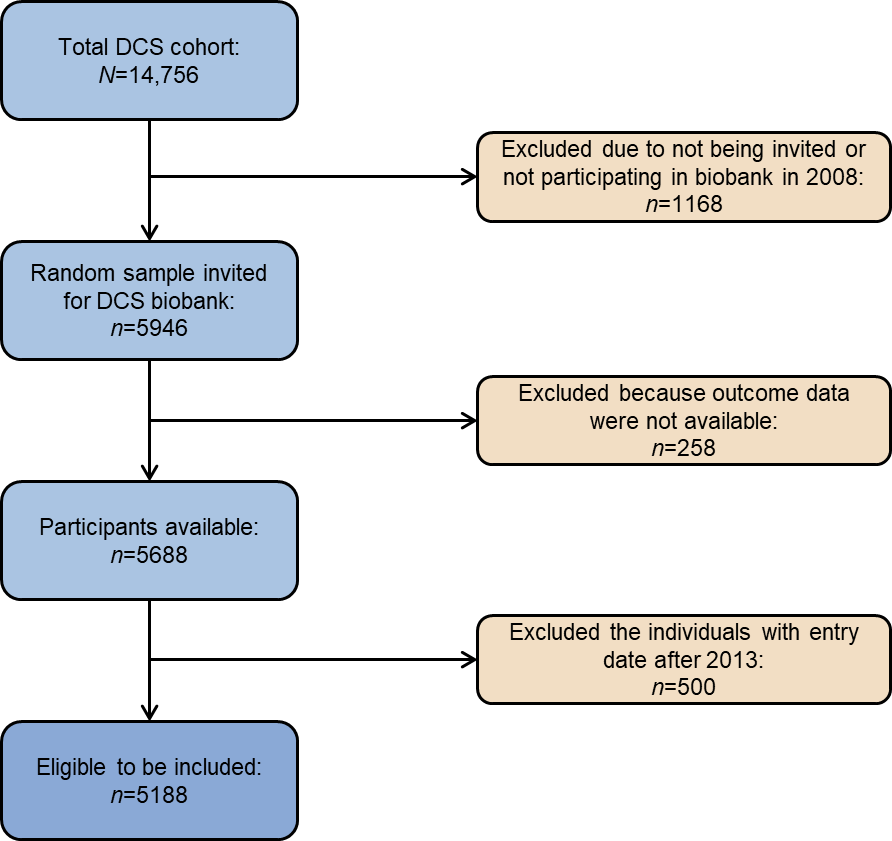


Figure S2: The flowchart of individual selection in Hoorn Diabetes Care System (DCS)

*Input data into MICADO*

MICADO works with input datafiles that organize risk factor and complication data in categories by five- year age groups and sex. For each risk factor, 3 or 8 classes of risk factor levels were used (Table S1), and up to 12 age groups ranging from 30-34 up to 85+ (Table S2). The history of complications was defined as yes/no, for each age group. Existing input files were based on Dutch primary care cohorts, which did not include the DCS cohort that was described above. For both observational datasets, new input files were created, showing the distribution over risk factor level categories and complications, by age and sex. There were few patients in some categories, especially at younger (below 50 years old) and elderly (higher than 75 years old) ages. When the sample size in an age category was too low (i.e. below 50 persons, and/or less than 5% of the total sample size of the respective dataset), that age category was merged with the following age category. This merging approach was performed for both risk factors and pre-existing events (Table S2). The aim of doing this was to increase the robustness of estimates.

Table S1: Classes of risk factors

| **Risk factor** | **Class 1** | **Class 2** | **Class 3** | **Class 4** | **Class 5** | **Class 6** | **Class 7** | **Class 8** |
| --- | --- | --- | --- | --- | --- | --- | --- | --- |
| **Smoking** | Never smoked | Current smoker | Stop smoking | - | - | - | - | - |
| **BMI** | < 25 | 25 to 29.9 | > 29.9 | - | - | - | - | - |
| **Cholesterol** | With medication  < 5.0 | With medication  5.0 to 6.4 | With medication  6.5 to 7.9 | With medication  > 8.0 | Without medication  < 5.0 | Without medication  5.0 to 6.4 | Without medication  6.5 to 7.9 | Without medication  > 8.0 |
| **SBP** | With medication  < 120 | With medication  120 to 139.9 | With medication  140 to 159.9 | With medication  > 159.9 | Without medication  < 120 | Without medication  120 to 139.9 | Without medication  140 to 159.9 | Without medication  > 159.9 |
| **HbA1c** | < 6.5% | 6.5% to 6.9% | 7.0 to 7.4% | 7.5% to 7.9% | 8.0% to 8.4% | 8.5% to 8.9% | 9.0% to 9.4% | > 9.4% |
| BMI: Body mass index; SBP: Systolic blood pressure; HbA1c: glycated haemoglobin | | | | | | | | |

Table S2: The age categories for men and women in CMS and DCS cohorts

| Cohort | Risk factor | Age category | | | | | | | | | | | |
| --- | --- | --- | --- | --- | --- | --- | --- | --- | --- | --- | --- | --- | --- |
|  |  | 30-34 | 35-39 | 40-44 | 45-49 | 50-54 | 55-59 | 60-64 | 65-69 | 70-74 | 75-79 | 80-84 | 85+ |
| CMS cohort | Smoking | Cat. 1 | | | | | Cat. 2 | | Cat. 3 | Cat. 4 | Cat. 5 | Cat. 6 | |
|  | BMI | Cat. 1 | | | | | Cat. 2 | | Cat. 3 | Cat. 4 | Cat. 5 | Cat. 6 | |
|  | Cholesterol | Cat. 1 | | | | | Cat. 2 | | Cat. 3 | Cat. 4 | Cat. 5 | Cat. 6 | |
|  | SBP | Cat. 1 | | | | | Cat. 2 | | Cat. 3 | Cat. 4 | Cat. 5 | Cat. 6 | |
|  | HbA1c | Cat. 1 | | | | | Cat. 2 | | Cat. 3 | Cat. 4 | Cat. 5 | Cat. 6 | |
|  | MI | Cat. 1 | | | | | Cat. 2 | | Cat. 3 | Cat. 4 | Cat. 5 | Cat. 6 | |
|  | CHF | Cat. 1 | | | | | Cat. 2 | | Cat. 3 | Cat. 4 | Cat. 5 | Cat. 6 | |
|  | Stroke | Cat. 1 | | | | | Cat. 2 | | Cat. 3 | Cat. 4 | Cat. 5 | Cat. 6 | |
| DCS cohort | Smoking | Cat. 1 | | | | Cat. 2 | Cat. 3 | Cat. 4 | Cat. 5 | Cat. 6 | Cat. 7 | Cat. 8 | |
|  | BMI | Cat. 1 | | | | Cat. 2 | Cat. 3 | Cat. 4 | Cat. 5 | Cat. 6 | Cat. 7 | Cat. 8 | |
|  | Cholesterol | Cat. 1 | | | | Cat. 2 | Cat. 3 | Cat. 4 | Cat. 8 | Cat. 6 | Cat. 7 | Cat. 8 | |
|  | SBP | Cat. 1 | | | | Cat. 2 | Cat. 3 | Cat. 4 | Cat. 8 | Cat. 6 | Cat. 7 | Cat. 8 | |
|  | HbA1c | Cat. 1 | | | | Cat. 2 | Cat. 3 | Cat. 4 | Cat. 8 | Cat. 6 | Cat. 7 | Cat. 8 | |
|  | MI | Cat. 1 | | | | Cat. 2 | Cat. 3 | Cat. 4 | Cat. 8 | Cat. 6 | Cat. 7 | Cat. 8 | |
|  | CHF | Cat. 1 | | | | | Cat. 2 | Cat. 3 | Cat. 4 | Cat. 5 | Cat. 6 | Cat. 7 | |
|  | Stroke | Cat. 1 | | | | | Cat. 2 | Cat. 3 | Cat. 4 | Cat. 5 | Cat. 6 | Cat. 7 | |

BMI: Body mass index; SBP: Systolic blood pressure; HbA1c: glycated haemoglobin MI: Myocardial infarction; CHF: Chronic heart failure

*Assessing transferability of MICADO*

Systematic approaches to assessing the transferability of economic evaluation studies have been developed over the past decades [6, 7]. Goeree and co-authors reviewed seven unique checklists, flow charts, criteria and tools to assess the geographic transferability of health technology assessments [6]. Common across the checklists, is the reliance on expert opinion to assess the transferability of a model and the need for any adjustments, rather than assessing the validity of model predictions against observed data after adjustments were made.

We reviewed the seven transferability checklists and tools for items referring specifically to decision models [6]. Table S3 lists the criteria used to determine whether a cost-effectiveness analysis is transferable (Column “Knock-out criteria”). We identified only two of the knock-out criteria related to decision models: “transparency and quality of the model” [8-11] and “capability of performing sensitivity analysis” [12] (highlighted in italic in Table S3). The MICADO model met the “Knock-out criteria” (see Table S3).

The checklist items referring to health economic decision models were identified as: age & sex of the modelled population, health status and severity of disease, life expectancy, complication rates, socio-economic and educational status (Table S3, column “Items related to HE decision models”). In order to assess the transferability of the model, we focused on adjusting these items in a series of discrete steps to make them relevant to the CMS and DCS cohort. We called each discrete step of adjustment a “adjustment step” (see Table 2 and Table S4). It was not possible to adjust diabetes complications-related mortality or the baseline risk for complications due to a lack of data in the CMS cohort. Socio-economic status and education were also not adjusted since these were not included as distinct characteristics in the MICADO model. We then evaluated the impact of each additional adjustment (adjustment step) on the accuracy of the model to predict predefined outcomes over time, i.e. validity of predictions. Thus, we aimed to assess transferability in an objective way rather than based on expert opinions.

Table S3: Characteristics of selected transferability checklists

| Author | Checklist/Decision Tool | Number of items | Knock-out criteria | Items related to HE decision models |
| --- | --- | --- | --- | --- |
| Heyland et al [12] | generalizability criteria from two aspects: clinical and system | 10 questions | Studies fulfill:   1. Comprehensive description of competing alternatives 2. Sufficient evidence of clinical effectiveness or efficacy 3. Appropriate identification, measurement and valuation of all important costs 4. *Appropriate sensitivity analysis* | - From clinical generalizability: Are the patients described in the analysis similar to those patients you see in your own setting? |
| Späth et al [13] | transferability indicators | 5 indicators | Studies fulfill:   1. Perspective of the study from national level 2. Comparison of two or more options 3. Description of the evaluated therapies 4. The assessed therapies and/or its comparators are used in the health system of interest | - Characteristics of the treated patient population |
| Welte et al [8] | transferability decision chart | 14 factors | Studies fulfill:   1. The relevant technology is not comparable to the one that shall be used in the decision country. 2. The comparator is not comparable to the one that is relevant to the decision country. 3. *The study (Including the decision model) does not possess an acceptable quality.* | - The case-mix of the target population, such as age, sex, race, education, co-morbidity, severity of disease and risk factors - Disease prevalence/incidence - Life expectancy |
| Boulenger et al [14] | transferability information checklist | 42 questions | Not explained | - Is the target population of the health technology clearly stated by the authors or when it is not can it be inferred by reading the article? - Are the population characteristics described? (e.g., age, sex, health status, socio-economic status, inclusion/exclusion criteria) - If a model is used, is it described in detail? - Are the origins of the parameters used in the model given? |
| Drummond et al [10] | steps for determining appropriate methods for adjusting cost-effectiveness information | 4 steps | Exclusion criteria:   1. If either the experimental technology or the comparator(s) are not relevant in the jurisdiction of interest. 2. *If the methodological quality of the studies (Including the decision model) doesn’t meet local standards, which is similar to Welte’s general ‘knock-out’ criteria.* 3. If the study population is different between jurisdictions. | - The case-mix of the target population, such as age, sex, race, education, co-morbidity, severity of disease and risk factors - Disease prevalence/incidence - Life expectancy |
| Turner et al [11] | transferability checklist | 5 domains | 1. Are the policy and research questions being addressed relevant to your questions? (Yes/No) 2. What is the language of this HTA report? Is it possible to translate this report into your language? (Yes/No) 3. *Is there a description of the health technology (Including the decision model) being assessed? (Judgment needed)* 4. Is the scope of the assessment specified? (Judgment needed) 5. Has the report been externally reviewed? (Judgment needed) 6. Is there any conflict of interest? (Judgment needed) 7. When was the work that underpins this report done? Does this make it out of date for your purposes? (Judgment needed) 8. Have the methods of the assessment been described in the HTA report? (Judgment needed) | Safety domain:   - Does the population described for eligibility match the population to which it is targeted in the target setting? - Are there any reasons to expect differences in complication rates (eg, epidemiology, genetic issues, health care system [quality of care, surveillance])?   Effectiveness (including efficacy) domain:   - Would you expect the baseline risk of patients within your own setting to be the same as the baseline risk of those patients considered within the HTA report for adaptation? (assuming that patients receive the same treatment and same comparator) |
| Antonanzas et al [9] | transferability index | 7 critical objectives  16 noncritical objectives | Critical objective factors:   1. The relevant parameters needed to calculate the ratio cost/effectiveness are given in the study. 2. *The quality of the study (Including the decision model) is acceptable (from 8 factors).* 3. The evaluated technology is used in the new health context (This factor will not be taken into account if the economic evaluation is carried out to obtain relevant information before the potential use of the technology in the new context). 4. The comparator is available or used in the new context. 5. Treatment and comparator data, as well as relevant epidemiological parameters for the technology, are valid in the new context. 6. The study perspective coincides with that used in the new context. | Critical objective factors:   - The relevant parameters needed to calculate the ratio cost/effectiveness are given in the study.   Noncritical subjective factors:   - The model connecting variables and parameters can be adapted to the new context. - Life expectancy is similar in both contexts. |

The italic items are referring to the decision model part for transferability.

Table S4: The relations of adjustment steps with transferability checklists

| Transferability item | MICADO parameters | Model’s default | | DCS data | CMS data | Related adjustment step |
| --- | --- | --- | --- | --- | --- | --- |
| Age & sex | 5 year age groups, men & women | | Source: Dutch population, Dutch GP registries (not DCS) 2007 | Available, 2008-2013 | Available, 2000 | Base case |
| Health status, severity of disease | Increasing the level of HbA1c | | Source: Dutch GP registries (not DCS) 2007  For risk factors: derived in MICADO from DM incidence rate and prevalence of risk factors in general population. Except for HbA1c: DCS+ZODIAC | Available, 2008-2013 and over time | Available, 2000 | All adjustment steps |
|  | Risk factors | |  |  |  | adjustment step 1 |
|  | Complications | |  |  |  | adjustment step 2 |
| Life expectancy | Mortality all causes, general population | | Source:  Statistics Netherlands  DCS+ZODIAC  GP registries | Not adjusted | Adjusted based on Italian data | adjustment step 3 |
|  | Excess mortality related to diabetes | |  | Not available data | Not available data | Not applicable |
|  | Case fatality related to MI, CHF and stroke | |  | Not available data | Not available data | Not applicable |
| Complication rates | Increasing the level of HbA1c in each year | | Dutch general practice registries and large Dutch cohort studies, in general population (Doetinchem, LASA) and Diabetes specific (ZODIAC, DCS) [15] | Not adjusted | Not adjusted | Not applicable |
| Socio-economic status, education | Not applicable in MICADO | | - | - | - | - |

DCS: Hoorn Diabetes Care System; CMS: Casale Monferrato Survey; GP: General practitioner; HbA1c: glycated haemoglobin; DM: Diabetes mellitus; ZODIAC: Zwolle Outpatient Diabetes project Integrating Available; CareCHF: Chronic heart failure, MI: Myocardial infarction; LASA: Longitudinal Aging Study Amsterdam

Table S5: Definition of cardiovascular complications

| **Outcome** | **ICD CODES** |
| --- | --- |
| Incidence of MI | ICD-9: 410 & 798  ICD-10: I21, I22, I23; R99 |
| Incidence of CHF | ICD-9: 428  ICD-10: I50 |
| Incidence of stroke | ICD-9: 430-438 |

CHF: Chronic heart failure; MI: Myocardial infarction

*Model validity*

The MICADO model was run for each adjustment step and per cohort from time of entry into DCS and CMS cohorts up to 10 years of follow up. Outcomes predicted by MICADO were recorded and consisted of cumulative incidence of mortality, MI, stroke, and CHF. In predicting incidence, only the first event after diagnosis was counted.

Model validity was performed by comparing MICADO predictions with the mean and 95% CI of the observed cumulative incidences in each cohort at 10 years of follow-up, i.e. “calibration-in-the-large”. MICADO was judged to be well calibrated for a particular outcome if the model simulated probability fell within the 95% CI of the probability estimated from the observed data.

We also estimated the model validity by comparing model outcomes in terms of event rates to the observed rates, for each adjustment step and each outcome, using the following measures:

| ${MAE}_{i}= \frac{1}{m}\sum_{j=1}^{m} \vert q_{j}-p_{j}\vert$ | (1) |
| --- | --- |
| ${RMSE}_{i}= \sqrt{\frac{1}{m}\sum_{j=1}^{m} {(q_{j}-p_{j})}^{2}}$ | (2) |
| ${MAPE}_{i}= \frac{1}{m}\sum_{j=1}^{m} \vert\frac{{q_{j-}p}_{j}}{q_{j}}\vert$ | (3) |

where${MAE}_{i}$ is mean absolute error, ${RMSE}_{i}$ is the root mean squared error, and ${MAPE}_{i}$ is the mean absolute percentage error. All formulas refer to adjustment step *i*, where $m$ is the number of years, $q_{j}$ is the observed mortality rate or cumulative event rate in year j and $p_{j}$ is the model predicted rate in year j. For all measures, a lower value reflects a better model validity. While *MAE* and *RMSE* are absolute values, *MAPE* relates the error to the size of the effect.

We also investigated model validity for various subgroups (see Table S6). This allowed investigating whether some subgroups were more amenable for transferability issues than others. For the subgroup analyses, a similar approach as for the total dataset was followed for each subgroup separately.

*Sensitivity analyses*

To test the robustness of our findings, the model simulations were repeated with risk factor transition rates set to different values. Hence, the transition probabilities across the different levels of risk factors (e.g. HbA1c, SBP, cholesterol and BMI) were assumed to be zero (i.e. risk factors were held constant through the simulation) or twice their model default values. The aim of these sensitivity analyses was to determine the influence of the risk factor transition rates on model outcomes. This is especially relevant for the Italian setting, with missing information on risk factor values during follow-up. We assessed model validity for each sensitivity analysis using the methods described above.

Table S6: Description of subgroups

| Subgroup | description |
| --- | --- |
| Sex | Male and female |
| Age at baseline | Up to 64.9 years old and 65 or older |
| BMI at baseline | Up to 24.9, 25 to 29.9 and 30 or higher |
| HbA1c at baseline | Up to 7.4% and 7.5% or higher |

BMI: Body mass index; HbA1c: Glycated haemoglobin

Table S7: The observed versus predicted and the measures of average prediction error over 10 years – base case adjustment step

| **Outcome** |  | |  | |  | | **CMS** | | |  | |  | | |  | |  | |  | |  | | **DCS** |  | |  | |  |
| --- | --- | --- | --- | --- | --- | --- | --- | --- | --- | --- | --- | --- | --- | --- | --- | --- | --- | --- | --- | --- | --- | --- | --- | --- | --- | --- | --- | --- |
|  | | **Observed** | | **95% CI** | | **Predicted** | | **MAE^*^** | **RMSE*** | | **MAPE^*^** | |  | **Observed** | | **95% CI** | | **Predicted** | | **MAE^*^** | | **RMSE*** | | | **MAPE^*^** | | | |
| **Overall mortality** | | 0.40 | | 0.37-0.42 | | 0.42 | | 0.05 | 0.15 | | 0.38 | |  | 0.26 | | 0.24-0.27 | | 0.35 | | 0.06 | | 0.20 | | | 0.70 | |  |  |
| **MI** | | 0.07 | | 0.06-0.08 | | 0.11 | | 0.03 | 0.09 | | 0.77 | |  | 0.03 | | 0.02-0.03 | | 0.11 | | 0.05 | | 0.15 | | | 2.58 | |  |  |
| **CHF** | | 0.12 | | 0.11-0.14 | | 0.07 | | 0.03 | 0.10 | | 0.44 | |  | 0.04 | | 0.04-0.05 | | 0.06 | | 0.01 | | 0.01 | | | 0.11 | |  |  |
| **Stroke** | | 0.10 | | 0.09-0.12 | | 0.06 | | 0.03 | 0.08 | | 0.42 | |  | 0.05 | | 0.04-0.06 | | 0.06 | | 0.01 | | 0.01 | | | 0.23 | | | |

CMS: Casale Monferrato Survey; DCS: Hoorn Diabetes Care System; CI: confidence interval; MAE: mean absolute error; RMSE: root mean square error; MAPE: mean absolute percentage error; MI: Myocardial infarction; CHF: Chronic heart failure

* Lower values mean a better fit.

Table S8: The observed versus predicted and the measures of average prediction error over 10 years – adjustment step 1

| **Outcome** |  | |  | |  | | **CMS** | | |  | |  | | |  | |  | |  | |  | | **DCS** |  | |  | |  |
| --- | --- | --- | --- | --- | --- | --- | --- | --- | --- | --- | --- | --- | --- | --- | --- | --- | --- | --- | --- | --- | --- | --- | --- | --- | --- | --- | --- | --- |
|  | | **Observed** | | **95% CI** | | **Predicted** | | **MAE^*^** | **RMSE*** | | **MAPE^*^** | |  | **Observed** | | **95% CI** | | **Predicted** | | **MAE^*^** | | **RMSE*** | | | **MAPE^*^** | | | |
| **Overall mortality** | | 0.40 | | 0.37-0.42 | | 0.42 | | 0.04 | 0.13 | | 0.35 | |  | 0.26 | | 0.24-0.27 | | 0.35 | | 0.06 | | 0.19 | | | 0.66 | |  |  |
| **MI** | | 0.07 | | 0.06-0.08 | | 0.12 | | 0.03 | 0.10 | | 0.85 | |  | 0.03 | | 0.02-0.03 | | 0.12 | | 0.05 | | 0.16 | | | 2.77 | |  |  |
| **CHF** | | 0.12 | | 0.11-0.14 | | 0.06 | | 0.04 | 0.12 | | 0.54 | |  | 0.04 | | 0.04-0.05 | | 0.05 | | 0.01 | | 0.01 | | | 0.16 | |  |  |
| **Stroke** | | 0.10 | | 0.09-0.12 | | 0.06 | | 0.03 | 0.08 | | 0.41 | |  | 0.05 | | 0.04-0.06 | | 0.06 | | 0.01 | | 0.02 | | | 0.26 | | | |

CMS: Casale Monferrato Survey; DCS: Hoorn Diabetes Care System; CI: confidence interval; MAE: mean absolute error; RMSE: root mean square error; MAPE: mean absolute percentage error; MI: Myocardial infarction; CHF: Chronic heart failure

* Lower values mean a better fit.

Table S9: The observed versus predicted and the measures of average prediction error over 10 years – adjustment step 2

| **Outcome** |  | |  | |  | | **CMS** | | |  | |  | | |  | |  | |  | |  | | **DCS** |  | |  | |  |
| --- | --- | --- | --- | --- | --- | --- | --- | --- | --- | --- | --- | --- | --- | --- | --- | --- | --- | --- | --- | --- | --- | --- | --- | --- | --- | --- | --- | --- |
|  | | **Observed** | | **95% CI** | | **Predicted** | | **MAE^*^** | **RMSE*** | | **MAPE^*^** | |  | **Observed** | | **95% CI** | | **Predicted** | | **MAE^*^** | | **RMSE*** | | | **MAPE^*^** | | | |
| **Overall mortality** | | 0.40 | | 0.37-0.42 | | 0.42 | | 0.04 | 0.13 | | 0.33 | |  | 0.26 | | 0.24-0.27 | | 0.35 | | 0.06 | | 0.19 | | | 0.65 | |  |  |
| **MI** | | 0.07 | | 0.06-0.08 | | 0.14 | | 0.04 | 0.13 | | 1.15 | |  | 0.03 | | 0.02-0.03 | | 0.13 | | 0.06 | | 0.18 | | | 3.23 | |  |  |
| **CHF** | | 0.12 | | 0.11-0.14 | | 0.08 | | 0.03 | 0.09 | | 0.42 | |  | 0.04 | | 0.04-0.05 | | 0.06 | | 0.01 | | 0.01 | | | 0.14 | |  |  |
| **Stroke** | | 0.10 | | 0.09-0.12 | | 0.06 | | 0.03 | 0.08 | | 0.43 | |  | 0.05 | | 0.04-0.06 | | 0.06 | | 0.01 | | 0.01 | | | 0.22 | | | |

CMS: Casale Monferrato Survey; DCS: Hoorn Diabetes Care System; CI: confidence interval; MAE: mean absolute error; RMSE: root mean square error; MAPE: mean absolute percentage error; MI: Myocardial infarction; CHF: Chronic heart failure

* Lower values mean a better fit.

Table S10: The observed versus predicted and the measures of average prediction error over 10 years – adjustment step 3

| **Outcome** |  | |  | |  | | **CMS** | | |  | |  | | |  | |  | |  | |  | | **DCS** |  | |  | |  |
| --- | --- | --- | --- | --- | --- | --- | --- | --- | --- | --- | --- | --- | --- | --- | --- | --- | --- | --- | --- | --- | --- | --- | --- | --- | --- | --- | --- | --- |
|  | | **Observed** | | **95% CI** | | **Predicted** | | **MAE^*^** | **RMSE*** | | **MAPE^*^** | |  | **Observed** | | **95% CI** | | **Predicted** | | **MAE^*^** | | **RMSE*** | | | **MAPE^*^** | | | |
| **Overall mortality** | | 0.40 | | 0.37-0.42 | | 0.38 | | 0.02 | 0.06 | | 0.20 | |  | 0.26 | | 0.24-0.27 | | 0.35 | | 0.06 | | 0.20 | | | 0.67 | |  |  |
| **MI** | | 0.07 | | 0.06-0.08 | | 0.15 | | 0.05 | 0.15 | | 1.27 | |  | 0.03 | | 0.02-0.03 | | 0.14 | | 0.06 | | 0.20 | | | 3.45 | |  |  |
| **CHF** | | 0.12 | | 0.11-0.14 | | 0.06 | | 0.04 | 0.11 | | 0.52 | |  | 0.04 | | 0.04-0.05 | | 0.05 | | 0.01 | | 0.01 | | | 0.16 | |  |  |
| **Stroke** | | 0.10 | | 0.09-0.12 | | 0.06 | | 0.03 | 0.08 | | 0.42 | |  | 0.05 | | 0.04-0.06 | | 0.06 | | 0.01 | | 0.01 | | | 0.24 | | | |

CMS: Casale Monferrato Survey; DCS: Hoorn Diabetes Care System; CI: confidence interval; MAE: mean absolute error; RMSE: root mean square error; MAPE: mean absolute percentage error; MI: Myocardial infarction; CHF: Chronic heart failure

* Lower values mean a better fit.

Table S11: The adjustment step with the minimum error over 10 years period

| **Outcome** |  | **CMS** |  |  |  | **DCS** |  |
| --- | --- | --- | --- | --- | --- | --- | --- |
|  | **MAE** | **RMSE** | **MAPE** |  | **MAE** | **RMSE** | **MAPE** |
| **Overall mortality** | 3 | 3 | 3 |  | 2 | 2 | 2 |
| **MI** | Base case | Base case | Base case |  | Base case | Base case | Base case |
| **CHF** | 2 | 2 | 2 |  | Base case | 3 | Base case |
| **Stroke** | 3 | 3 | 1 |  | 2 | 2 | 2 |

DCS: Hoorn Diabetes Care System; CMS: Casale Monferrato Survey; MAE: Mean absolute error; RMSE: Root mean square error; MAPE: Mean absolute percentage error; MI: Myocardial infarction; CHF: Chronic heart failure

*Results of subgroup analyses*

For mortality, the results for different sub-groups are shown in Figures S3 (CMS) and S4 (DCS) of the supplementary material. Table S12 shows the best fit in each subgroup for both cohorts. For both cohorts, the best fit for subgroups based on sex, BMI and HbA1c were in women, BMI lower than 25, and hbA1c higher than 7.5%, respectively. The best fit was obtained for people older than 65 years in the CMS cohort, whereas in the DCS cohort the model performed better for younger people (lower than 65). For CMS the predicted mortality rate for all subgroups tested was either a good fit or underpredicted, in DCS the model overpredicted the mortality rate, similar to the results for the complete cohort.

*Results of sensitivity analyses*

Figures S5 and S6 in the Supplementary materials show the fit of MICADO after changing the risk factor transition rates in MICADO and re-running the simulations. Overall, changes to transition rates had limited effect on the model predictions for mortality.

Figure S3: The observed mortality vs. the predicted by model for the sub-groups of CMS cohort

DCS: Hoorn Diabetes Care System; CMS: Casale Monferrato Survey;

Figure S4: The observed mortality vs. the predicted by model for the sub-groups of DCS cohort

DCS: Hoorn Diabetes Care System; CMS: Casale Monferrato Survey;

Figure S5: Observed mortality vs. the predicted by the model for the CMS cohort with risk transitions forced to zero and doubled; Left: With default mortality parameter, right: With updated mortality parameter (adjustment step 3)

CMS: Casale Monferrato Survey

Figure S6: The observed mortality vs. the predicted by model for the DCS cohort with risk transitions forced to zero and doubled; Left: With default mortality parameter, right: With updated mortality parameter (adjustment step 3)

DCS: Hoorn Diabetes Care System

Table S12: Best fit for each subgroup base on MAPE.

| **Subgroup** | **Best fit** |  |
| --- | --- | --- |
|  | CMS cohort | DCS cohort |
| **Sex** | Women | Women |
| **Age** | Higher than 65 | Lower than 65 |
| **BMI** | Lower than 25 | Lower than 25 |
| **HbA1c** | Higher than 7.5% | Higher than 7.5% |

BMI: Body mass index; HbA1c: glycated haemoglobin

1. Bruno, G., et al., *A population-based prevalence survey of known diabetes mellitus in Northern Italy based upon multiple independent sources of ascertainment.* Diabetologia, 1992. **35**(9): p. 851-856.

2. Pagano, E., et al., *Prediction of mortality and macrovascular complications in type 2 diabetes: validation of the UKPDS Outcomes Model in the Casale Monferrato Survey, Italy.* Diabetologia, 2013. **56**(8): p. 1726-1734.

3. Van Der Heijden, A.A., et al., *The Hoorn Diabetes Care System (DCS) cohort. A prospective cohort of persons with type 2 diabetes treated in primary care in the Netherlands.* BMJ open, 2017. **7**(5).

4. van’t Riet, E., et al., *The diabetes pearl: diabetes biobanking in The Netherlands.* BMC Public Health, 2012. **12**(1): p. 949.

5. Pagano, E., et al., *Prediction of mortality and major cardiovascular complications in type 2 diabetes: external validation of UKPDS outcomes model version 2 in two European observational cohorts.* Diabetes, Obesity and Metabolism, 2020.

6. Goeree, R., et al., *Transferability of health technology assessments and economic evaluations: a systematic review of approaches for assessment and application.* ClinicoEconomics and outcomes research: CEOR, 2011. **3**: p. 89.

7. Wijnen, B.F., et al., *How to prepare a systematic review of economic evaluations for informing evidence-based healthcare decisions: data extraction, risk of bias, and transferability (part 3/3).* Expert review of pharmacoeconomics & outcomes research, 2016. **16**(6): p. 723-732.

8. Welte, R., et al., *A decision chart for assessing and improving the transferability of economic evaluation results between countries.* Pharmacoeconomics, 2004. **22**(13): p. 857-876.

9. Antonanzas, F., et al., *Transferability indices for health economic evaluations: methods and applications.* Health economics, 2009. **18**(6): p. 629-643.

10. Drummond, M., et al., *Transferability of economic evaluations across jurisdictions: ISPOR Good Research Practices Task Force report.* Value in health, 2009. **12**(4): p. 409-418.

11. Turner, S., et al., *The health technology assessment adaptation toolkit: description and use.* International journal of technology assessment in health care, 2009. **25**(S2): p. 37-41.

12. Heyland, D.K., et al., *Economic evaluations in the critical care literature: do they help us improve the efficiency of our unit?* Critical care medicine, 1996. **24**(9): p. 1591-1598.

13. Späth, H.-M., et al., *Analysis of the eligibility of published economic evaluations for transfer to a given health care system: methodological approach and application to the French health care system.* Health Policy, 1999. **49**(3): p. 161-177.

14. Boulenger, S., et al., *Can economic evaluations be made more transferable?* The European Journal of Health Economics, 2005. **6**(4): p. 334-346.

15. Baan, C., G. Bos, and M. Jacobs-van der Bruggen, *Modeling chronic diseases: the diabetes module. Justification of (new) input data.* RIVM rapport 260801001, 2005.
